# The complete mitochondrial genome of the White-browed Tit-warbler

**DOI:** 10.1080/23802359.2022.2080010

**Published:** 2022-06-07

**Authors:** Hao Gao, Jiali Zhang, Ji Huang, Zhenyu Jin, Fuyong Zhao, Shaobin Li

**Affiliations:** a College of Life Science, Yangtze University, Jingzhou, China; b College of Agriculture, Yangtze University, Jingzhou, China

**Keywords:** Alpine bird, bushtit, *Leptopoecile*, mitochondrial genome, Tibet Plateau

## Abstract

The White-browed Tit-warbler (*Leptopoecile sophiae*) is a tiny and infrequently-seen bird species that is mainly distributed in alpine scrub and forests. This species is poorly understood with respect to its natural history and genetic information. In this paper, we first presented the whole mitochondrial genome of the White-browed Tit-warbler. The whole mitochondrial sequence of this species was determined by long-range PCR and conserved primer walking approaches. The results revealed that the whole mitochondrial genome of *L. sophiae* was 17,803 bp in length with 54.3% A + T content; the genome has the same gene order as that of other passerine birds, including 13 protein-coding genes, 2 rRNA genes, 22 tRNA genes and 1 non-coding control region. The control region (D-loop) of *L. sophiae* was located between tRNA-Gln and tRNA-Phe with 1155 bp length. Phylogenetic analysis indicated that the genome of *Leptopoecile* species is more closely related to *Aegithalos* species than to *Phylloscopus* species. These data provide value for further studies on molecular evolution and conservation genetics on these poorly-known *Leptopoecile* species at high altitudes.

The White-browed Tit-warbler (*Leptopoecile sophiae* Severtzov 1873) is a small purplish-blue bird (ca 8 g, infrequently seen) that commonly occurs in southwest China and the area around Himalayas and Tien Shan Mountains (Zheng 2005; BirdLife International 2021). Their typical breeding habitats are alpine shrub and high-altitude forest, and their altitudinal range varies from 2000 m to *ca*. 5000 m altitude (Lu et al. 2009; Harrap 2020; Gill et al. 2021). The genus *Leptopoecile* includes only two species (*L. sophiae* and Crested Tit-warbler *L. elegans*), both of which are still poorly understood, particularly with regards to their natural histories that are currently based on simple descriptions (Lu et al. 2009) or species accounts (e.g. Zheng 2005; Harrap 2020). In this study, we describe the whole mitochondrial genome of *L. sophiae* for the first time. The newly-sequenced whole mitochondrial genome will provide fundamental data for further studies on this poorly-known genus.

A blood sample was collected by puncturing the brachial vein of a female White-browed Tit-warbler (ID: ls_2101) that was caught in mist nets, on 17 June 2021 near the Buha river at Tianjun County (37.32°N, 99.01°E; 3410 m above the sea level), Qinghai Province. Adult birds were captured and blooded under the Wild Animal Conservation Law of China. Bird ringing was permitted by The National Bird Combination Center. Blood was stored in lysis buffer and genome DNA was extracted from blood with TIANamp Genomic DNA kits (details see Li et al. 2018). The remaining blood sample is now stored in the herbarium room 317 of #1 Teaching Building at West campus of Yangtze University (www.yangtzeu.edu.cn, Dr. Shaobin Li, shaobinlee@126.com). The entire sequence of the White-browed Tit-warbler mitochondrial genome was determined by 11 pairs of conserved primers (P1F-GCATGGCACTGAAGATGCCA, P1R-GGCTTAAAGAGGGCTCGATTG; P2F-AAGACAGGTCAAGGTATAGCC, P2R-CTCTCGGAGGAGATTGCGCTG; P3F-CTTACAGGATACTGGTTCGCA, P3R-CGATAGCTTATTTAGCTGAC; P4F-GTCACTATGATAAAGTGAAC, P4R-CGAAGATTAGGTACAGAGTG; P5F-CGATAAGAAGAGGAATTAAACCTC, P5R-TATGGGGGTTCGACTCCTTCC; P6-ATGCCACGACGATACTCAGA, P6R-GTCATAGAGGGAAGGCTAG; P7F-AAGGCCATAAATGAGCCCT, P7R-CTGTCTTGGTTAGACTAAC; P8F-CCTAGAAATTGCACTTCTTCTCC, P8R-GGTCCATTACTTTCACTTGG; P9F-CTGCTAACTCTTGCATCTGAG, P9R-TGATGGTGTAGGGAGGTCG; P10F-CACTCCGGCCTAATCAAAGC, P10R-CTTTGGTTTACAAGACCAATG; P11F-CACCCATTCATCATCATCGGAC, P11R-GCTAGACGTCTTGGGCTACC). PCR protocols were according to Li et al. (2018). The PCR products were sequenced directly, or if needed first cloned into a pMD18-T vector (Takara, JAP) and then sequenced. After quality-proofing of the obtained fragments, the complete mitochondrial genome sequence was assembled manually using DNAstar v7.1 software (Burland 2000).

Our results show that the entire mitochondrial genome of the White-browed Tit-warbler comprises of 17,803 bp nucleotides in length, which exhibit the typical mitochondrial structure of passerine birds (Cao et al. 2017; Qin et al. 2019), including 13 protein-coding genes, 22 tRNA genes, 2 rRNA genes and a non-coding control region. The overall nucleotide composition includes A (29.87%), C (31.44%), G (14.28%) and T (24.41%), with a total A and T content of 54.28%. The entire mitochondrial sequence has been deposited in GenBank with accession number of MZ677204. The H-strand contains 28 of the 37 genes and the remainders on the L-strand. Of the 13 protein-coding genes of the *L. sophiae* mitochondrial genome, all of them use ATG as the start codon (except for *Cox*I using GTG). With respect to terminate codon, most of them use TAA or TAG. The control region of *L. sophiae* comprise 1155 bp in length between two tRNAs (tRNA-Gln and tRNA-Phe), which included a number of conserved sequences that is considered important in the replication and transcription of mitochondrial genome.

Phylogenetic analyses were conducted with whole mitochondrial data of this study and 27 other bird species from the GenBank database. The topology of the tree was inferred using the maximum likelihood method in the program MEGA 7 (Kumar et al. 2016). Execution model was statistically well supported by high bootstrap values at most nodes (Figure 1). The phylogenetic tree revealed that *Leptopoecile* species (e.g. *L. sophiae*) were more closely related to *Aegithalos* species than *Phylloscopus* species (genome identity: 87.1% vs. 85.3%). All the clades were consistent with the traditional morphology-based taxonomies and recent molecular taxonomies (Zheng 2005; Gill et al. 2021). At present, natural history and genetic information for all the *Leptopoecile* species are still poorly understood (Xiao et al. 2017; Gill et al 2021), and no complete mitochondrial genome of *Leptopoecile* species has been reported in Genebank (Figure 1). Hence, the complete mitochondrial genome presented in the present study could now provide valuable information for further studies on phylogenetic studies and conservation genetics on these poorly-known bushtits. 

**Figure 1.**
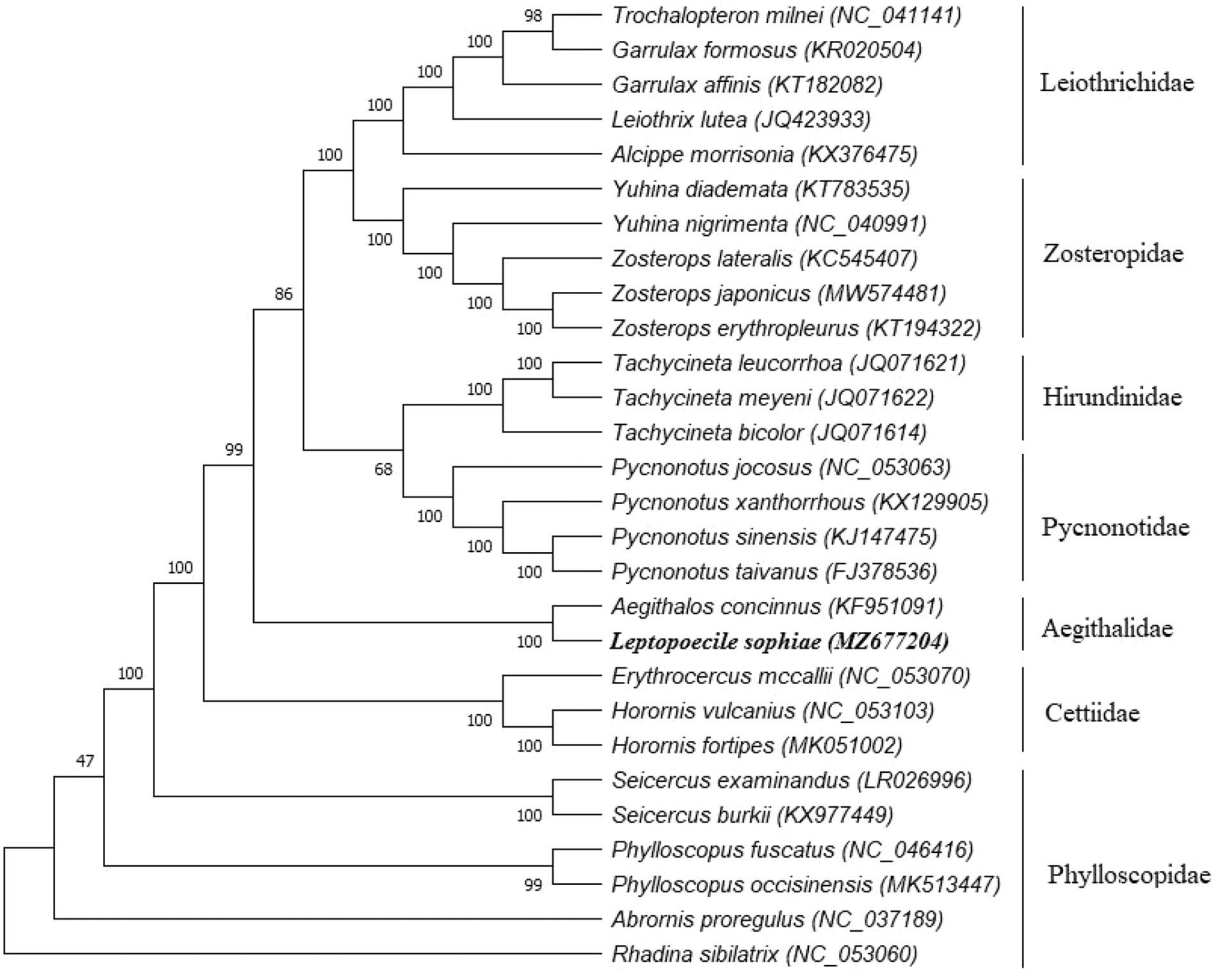
Phylogenetic relationships of 28 taxa were inferred using the maximum likelihood method based on their entire mitochondrial genome (their genbank accession number in parentheses; numbers at branches denote bootstrap values from 1000 replications; the sequence from this study is marked in bold).

## Data Availability

The genome sequence data that support the findings of this study are openly available in GenBank of NCBI at [https://www.ncbi.nlm.nih.gov] (https://www.ncbi.nlm.nih.gov/) under the accession no. MZ677204.
